# Cognitive Impairment in Myotonic Dystrophy Type 1 Is Associated with White Matter Damage

**DOI:** 10.1371/journal.pone.0104697

**Published:** 2014-08-12

**Authors:** Francesca Caso, Federica Agosta, Stojan Peric, Vidosava Rakočević-Stojanović, Massimiliano Copetti, Vladimir S. Kostic, Massimo Filippi

**Affiliations:** 1 Neuroimaging Research Unit, Institute of Experimental Neurology, Division of Neuroscience, San Raffaele Scientific Institute, Vita-Salute San Raffaele University, Milan, Italy; 2 Neurology Clinic, School of Medicine, University of Belgrade, Belgrade, Serbia; 3 Biostatistics Unit, IRCCS-Ospedale Casa Sollievo della Sofferenza, Foggia, Italy; University of Ulm, Germany

## Abstract

**Objective:**

To investigate grey (GM) and white matter (WM) abnormalities and their effects on cognitive and behavioral deficits in a large, phenotypically and genotypically well-characterized cohort of classic adult (aDM1, age at onset ≥20 years) or juvenile (jDM1, age at onset <20 years) patients with myotonic dystrophy type 1 (DM1).

**Methods:**

A case-control study including 51 DM1 patients (17 jDM1 and 34 aDM1) and 34 controls was conducted at an academic medical center. Clinical, cognitive and structural MRI evaluations were obtained. Quantitative assessments of regional GM volumes, WM hyperintensities (WMHs), and microstructural WM tract damage were performed. The association between structural brain damage and clinical and cognitive findings was assessed.

**Results:**

DM1 patients showed a high prevalence of WMHs, severe regional GM atrophy including the key nodes of the sensorimotor and main cognitive brain networks, and WM microstructural damage of the interhemispheric, corticospinal, limbic and associative pathways. WM tract damage extends well beyond the focal WMHs. While aDM1 patients had severe patterns of GM atrophy and WM tract damage, in jDM1 patients WM abnormalities exceeded GM involvement. In DM1, WMHs and microstructural damage, but not GM atrophy, correlated with cognitive deficits.

**Conclusions:**

WM damage, through a disconnection between GM structures, is likely to be the major contributor to cognitive impairment in DM1. Our MRI findings in aDM1 and jDM1 patients support the hypothesis of a degenerative (premature aging) origin of the GM abnormalities and of developmental changes as the principal substrates of microstructural WM alterations in DM1.

## Introduction

Myotonic dystrophy type 1 (DM1) is a dominantly inherited, multisystem progressive disease, caused by a trinucleotide (CTG) expansion in the 3′-untranslated region of the dystrophia myotonica protein kinase gene on chromosome 19 [1]. DM1 is the most common form of adult-onset muscular dystrophy and is characterized by limb muscle weakness, myotonia, and multiorgan involvement, including cataract, cardiac conduction defects, insulin resistance, gonadal atrophy, and central nervous system (CNS) pathology [1].

Impaired mental functions have been reported in DM1 patients, predominantly in the congenital disease form [2]. Daytime sleepiness, fatigue, executive and visuospatial dysfunctions, and anxious personality traits (deteriorating with age) are common manifestations in patients with the classic adult (aDM1, age at onset ≥20 years) or juvenile (jDM1, age at onset <20 years) forms of DM1 [2]. CNS pathological abnormalities in DM1 patients consist of white matter (WM) rarefaction, dilated Virchow-Robin spaces and mild gliosis are described [3]–[5]. Intraneuronal neurofibrillary tangles (NFT) of the type seen in frontotemporal dementia, without plaques, have been demonstrated in the DM1 brains [3]–[5], connecting this disorder to a subset of neurodegenerative diseases termed tauopathies.

In these patients, routine brain MRI often shows nonspecific pathological findings such as white matter hyperintensities (WMHs), ventricular enlargement and brain atrophy [6]–[13]. Studies using perfusion [14] or 18F-deoxy-glucose positron emission tomography demonstrated hypoperfusion/hypometabolism of frontal and temporal lobes in patients with DM1 [15]. More recently, brain involvement has been demonstrated using advanced MR techniques in small samples of DM1 patients. Grey matter (GM) atrophy has been shown in various cortical regions, basal ganglia and thalami [15]–[19]. A distributed white matter (WM) involvement has been found by a few studies using diffusion tensor (DT) MRI [8], [17]–[21]. WM abnormalities have been detected even without corresponding lesions on T2-weighted images, thus suggesting microstructural alterations. Several reports assessed correlations of brain morphological changes with clinical and cognitive features in DM1, with conflicting results [2]. DT MRI findings have been related to disease duration, CTG repeat expansion sizes, and disease severity scales [18]. A relationship between the extent of WMHs and cognitive deficits has been suggested [6], [7], [10], [12], [13], [15], while no association was found between WM microstructural damage and cognitive impairment [18].

The goal of this study was to investigate GM and WM abnormalities and their effects on clinical and cognitive deficits in a large, phenotypically and genotypically well-characterized cohort of noncongenital DM1 patients. We also assessed separately jDM1 and aDM1 cases in order to evaluate the influence of age and disease duration on brain structural damage.

## Materials and Methods

### Subjects

DM1 patients were enrolled consecutively from the population attending the Neurology Clinic, Clinical Centre of Serbia, University of Belgrade, Serbia. The diagnosis of DM1 was based on clinical examination, electromyography and Southern blot analysis showing the CTG repeat expansion on chromosome 19. Patients were excluded if they had: congenital DM1; traumatic brain injury history; intracranial mass; other major medical, neurological or psychiatric illnesses; and/or any contraindication to MRI. Sixty-four DM1 patients accepted to participate into the study. One patient did not perform MRI due to an implantable pacemaker. MRI scans from 12 patients were excluded because of major strokes, brain malformations or movement artifacts. Therefore, 51 DM1 patients were included into the analysis. Thirty-four age and sex-matched healthy controls (HC) were studied.

DM1 patients underwent a detailed neurological evaluation consisting in history collection and assessment of muscle involvement using the Muscular Impairment Rating Scale (MIRS) [22], sleepiness using the Daytime Sleepiness Scale (DSS) [23], and fatigue using the Krupp’s Fatigue Severity Scale (KFSS) [24]. Approval was received from the Ethics committee on human experimentation of the School of Medicine, University of Belgrade, and written informed consent was obtained from all subjects.

### Genetic assessment

Blood samples were obtained from all patients and DNA was purified from peripheral blood leukocytes. Molecular analysis of CTG triplet repeat expansions included small pool PCR analysis using serial dilutions of EcoR1 digested genomic DNA (50–500 pg). The products were detected by Southern blotting with subsequent hybridization to the end-labeled (CTG) 12 oligonucleotide probe. Sizes of alleles were determined by plotting band sizes against an exponential curve determined by a co-electrophoresed DNA size marker using TotalLab 1.10 program.

### Cognitive evaluation

Cognitive functions were explored by an experienced neuropsychologist blinded to MRI results, as previously described [25]. Briefly, global cognitive status was assessed using the Addenbrooke’s Cognitive Examination–Revised (ACE-R) [26]; reasoning and problem solving with the Raven progressive matrices [27], and the Wechsler-Adult Intelligence Scale (WAIS) similarities, digit symbol coding and arithmetic subtests [28]; attention with the ACE-R orientation and attention subscore [26]; executive functions with the ACE-R fluency subscore [26], phonemic and semantic fluency tests [28], Trail Making Test (TMT) [29] and Wisconsin card sorting test (WCST) [29]; memory with the WAIS digit span [28], ACE-R memory subscore [26], Rey Auditory Verbal Learning test (RAVLT) [30], and Rey-Osterrieth complex figure (ROCF) recall [29]; visuospatial abilites with the ACE-R visuospatial subscore [26], ROCF Copy Test [29], WAIS block design [28], and Hooper Visual Organization Test (VOT) [31]; and language with the ACE-R language subscore [26] and Boston Naming Test (BNT) [32]. Results were considered abnormal if subjects performed at least 1.0 standard deviation below the normative mean score of healthy population according to the appropriate references, when available. Adjusted WAIS scores <6 were considered abnormal. There is no standardization of the ACE and Hooper VOT tests on Serbian adult population; as a consequence, the frequencies of pathological scores at such tests were not calculated. Mood and behavioral disorders were examined using the Hamilton Depression (HDRS) [33] and Anxiety Rating (HARS) [34] scales. HDRS score ≥17 indicated moderate or severe depression, while HARS score ≥18 indicated significant anxiety.

### MRI study

MRI scans were acquired using a 1.5 T system (Philips Medical Systems, Achieva). Brain dual-echo (DE) turbo spin-echo, 3D T1-transient field echo, and pulsed gradient SE single shot echo-planar sequences were obtained. All MRI post-processing was performed by a single experienced observer, blinded to subject’s identity. The following MRI variables were assessed: a) WMH severity and location using a visual scale (Wahlund scale) [35], a semi-automatic threshold-based approach (WMH load), and lesion probability maps; b) the pattern of GM atrophy using voxel-based morphometry (VBM) in SPM8; and c) WM mean diffusivity (MD), fractional anisotropy (FA), radial diffusivity (radD), and axial diffusivity (axD) using tract-based spatial statistics (TBSS) in FSL. The Appendix S1 reports the complete MRI protocol and details on the MRI analysis.

### Statistical analysis

Demographic and clinical variables were compared between groups using the Pearson chi-square for categorical variables and the Mann-Whitney U test for continuous variables. Cognitive scores were converted as means (and ranges) or frequencies (and percentages) of subjects with pathological scores; between-group comparisons were performed using the Poisson model for continuous variables and the Pearson chi-square for categorical variables with false-discovery rate correction for multiple comparisons (with and without adjusting for subjects’ age). Correlations between WMH load and demographic, clinical and neuropsychological variables were assessed using the Spearman coefficient, also adjusting for subject’s age. P<0.05 was considered for statistical significance. All analyses were performed using SAS Release 9.1.3 (SAS Institute).

Using SPM8, GM volume differences were tested using analyses of covariance adjusted for age and total intracranial volume (TICV). The level of significance was set at p<0.05, family-wise error (FWE)–corrected for multiple comparisons. DT MRI voxelwise statistics were performed using a permutation-based inference tool for nonparametric statistical thresholding (“randomize”, part of FSL), adjusting for subject’s age [36]. The number of permutations was set at 5000 [36]. The resulting statistical maps were thresholded at p<0.05 corrected for multiple comparisons at a cluster level using the threshold-free cluster enhancement option [37]. Analyses were tested also adjusting for years of education.

To assess whether clinical, cognitive and conventional MRI variables were associated with GM atrophy and microstructural WM damage, regression models in SPM8 (adjusted for age and TICV) and FSL (adjusted for age) were run (p<0.05, corrected for multiple comparisons). To minimize the number of comparisons, axD and radD were not included in the correlation analysis.

## Results

### Demographic and clinical findings

Table 1 shows the main demographic and clinical findings of DM1 patients and healthy controls (HC). There were 17 jDM1 and 34 aDM1 cases. Compared with HC, all patients had lower education and jDM1 patients were younger. By definition, jDM1 were younger with earlier disease onset compared with aDM1 patients. The two patient groups had similar CTG repeat numbers. DM1 patients showed a moderate muscular impairment and 40% of them had mild to moderate sleepiness. Only 4 jDM1 and 8 aDM1 patients had a forced vital capacity (FVC) <70%, with no relationship between DSS score and FVC. In the whole DM1 group, 52% of patients showed mild fatigue but when we considered the two groups separately the percentage of patients with fatigue raised up to 64% in aDM1 in contrast to 24% in jDM1 (p = 0.03, age-corrected p = 0.17). No further differences in demographic and clinical findings were found between the two patient groups.

**Table 1:** Main demographic, clinical and conventional MRI data from healthy controls and DM1 patients.

	HC	DM1	jDM1	aDM1	p
**Number**	34	51	17	34	-
**Women/men**	21/13	24/27	5/12	19/15	jDM1 *vs* HC: p = 0.04
**Age at MRI** **[years]**	45±10(24–61)	42±10(19–64)	34±9(19–46)	46±9(24–64)	jDM1 *vs* HC: p = 0.003 jDM1 *vs* aDM1: p = 0.001
**Education** **[years]**	13.5±2.5(8–17)	11±1.8(8–15)	11±1.1(8–12)	10±2.1(8–15)	all DM1 *vs* HC: p<0.001
					jDM1 *vs* HC: p<0.001
					aDM1 *vs* HC: p<0.001
**Age at onset** **[years]**	-	22.8±9.3(3–47)	12.7±4.5(3–19)	27.8±6.7(20–47)	jDM1 *vs* aDM1: p<0.001
**Disease duration** **[years]**	-	19.2±8.5(2–41)	21.7±9.7(9–41)	17.9±7.8(2–33)	ns
**CTG repeats**	-	750±276(177–1534)	777±352(182–1534)	737±240(177–1319)	ns
**MIRS,** **minimum 1-** **maximum 5**	-	3.3±0.7(2–5)	3.1±0.7(2–4)	3.4±0.8(2–5)	ns
**KFSS, cut off:** **36**	-	34.8±14.3(52%)	30.9±14(29%)	36.8±14.5(64%)	ns
**DSS, cut** **off: 6**	-	6.5±3.5(40%)	6.0±3.6(35%)	6.5±3.2(42%)	ns
**Whalund** **scale score**	0.3±0.8(0–3)	3.6±2.5(0–8)	2.8±1.9(0–6)	4.1±2.6(0–8)	all DM1 *vs* HC: p<0.001
					jDM1 *vs* HC: p<0.001
					aDM1 *vs* HC: p<0.001
**WMH** **load [ml]**	0.05±0.2(0–1)	2.0±2.5(0–9.1)	1.2±1.5(0–4.9)	2.5±2.8(0–9.1)	all DM1 *vs* HC: p<0.001
					jDM1 *vs* HC: p<0.001
					aDM1 *vs* HC: p^3^<0.001

Values are means ± standard deviations (range or % of subjects with pathological scores relative to cut off values) or number of subjects. P values refer to Pearson chi-square or Mann-Whitney U test (see text for further details). Abbreviations: aDM1, Myotonic dystrophy type 1 with adult onset (≥20 years); DM1, Myotonic dystrophy type 1; DSS, Daytime Sleepiness Scale; KFSS, Krupp’s Fatigue Severity Scale; HC, Healthy controls; jDM1, Myotonic dystrophy type 1 with childhood onset (<20 years); ns, not significant; MIRS, Muscular impairment rating scale; MRI, magnetic resonance imaging; WMH, white matter hyperintensity.

### Neuropsychological findings

Table 2 shows the cognitive findings in DM1 patients. The most involved cognitive domains in DM1 patients were visuospatial abilities, executive functions, reasoning and naming. Verbal memory was relatively preserved compared with visuospatial memory. No patient met the DSM-IV criteria for major psychiatric illness.

**Table 2:** Neuropsychological and behavioural data of DM1 patients.

	DM1	jDM1	aDM1
**Cognitive Screening**			
ACE-R global score	83.3±8.1	86.4±6.4	81.4±8.7
**Reasoning and problem solving**			
Raven matrices	25.7±10.9 (57%)	28.9±11.7 (56%)	23.8±10.3 (58%)#
WAIS, similarities	7.8±2.3 (19%)	8.8±2.2 (6%)	7.3±2.3 (27%)
WAIS, digit symbol coding	6.8±1.9 (22%)	7.5±1.2 (0%)	6.5±2.2 (33%)
WAIS, arithmetic	6.7±2.5 (40%)	6.4±2.7 (50%)	6.8±2.5 (33%)
**Orientation and attention**			
ACE-R, orientation & attention	15.7±1.8	15.7±1.9	15.8±1.9
**Memory**			
WAIS, Digit Span	7.8±2.7 (11%)	8.9±3.3 (12%)	5.6±2.5 (10%)
ACE-R, memory	22.8±3	23.4±2.6	22.5±3.3
RAVLT, total score	46.9±7.9 (22%)	48.8±6.8 (12%)	45.9±8.5 (27%)
RAVLT, recognition	12.9±2.4 (35%)	13±1.6 (44%)	12.8±2.8 (33%)
Rey’s Figure, recall	13.1±5.2 (47%)	15.5±5 (44%)	12.1±5 (48%)#
**Visuospatial abilities**			
ACE-R, visuospatial	13.3±2.1	14.1±2	12.8±2.1
Rey’s Figure, copy	23.8±5.5 (75%)	24.9±4.6 (69%)	23.4±6 (79%)
WAIS, block design	5.5±2.3 (47%)	5.4±2.1 (56%)	5.6±2.5 (42%)
Hooper VOT	14.1±4.9	16.1±3.7	12.8±5.3#
**Executive functions**			
ACE-R, fluency	8.6±3.5	9.9±2.9	7.9±3.8
Phonemic fluency	27.2±7.9 (26%)	31.3±10.5 (12%)	25.2±5.7 (33%)# *
Category fluency	17.4±4.1 (10%)	18.8±3.6 (6%)	16.8±4.3 (12%)
TMT, part A	55.6±21.6 (39%)	47.3±15.8 (25%)	59.7±23.3 (45%)#
TMT, part B	156.6±82.8 (58%)	139.6±96.1 (44%)	166±76.3 (66%)#
WCST, categories	2.6±2.2 (67%)	3.2±2.4 (62%)	2.3±2.1 (69%)
WCST, perseverative responses	28.1±20.3 (49%)	27.7±19.8 (50%)	28.4±21.3 (48%)
WCST, maintaining set inability	0.6±0.9 (9%)	0.7±0.9 (6%)	0.6±0.9 (10%)
**Language**			
ACE-R, language	22.7±2.9	23.4±2.9	22.3±2.9
BNT	49.1±5.5 (62%)	52.6±3.7 (44%)	47.2±5.6 (72%)#
**Mood & Behaviour**			
HDRS	11.1±6.3 (20%)	9.2±4.4 (12%)	12±7.1 (23%)#
HARS	10.6±5.5 (14%)	8.9±3.8 (0%)	11.4±6.2 (21%)#

Values are means ± standard deviations (% of subjects with abnormal test score compared with normative data of reference, see text for further details).

# p<0.05 in aDM1 *vs* jDM1 patients (Poisson model, false-discovery rate adjusted for multiple comparisons;

*age-adjusted p values). Abbreviations: ACE-R, Addenbrooke’s Cognitive Examination–Revised; aDM1, Myotonic dystrophy type 1 with adult onset (≥20 years); BNT, Boston Naming Test; DM1, Myotonic dystrophy type 1; HDRS, Hamilton Depression Rating Scale; HARS, Hamilton Anxiety Rating Scale; jDM1, Myotonic dystrophy type 1 with early onset (<20 years); ns, not significant; RAVLT, Rey Auditory Verbal Learning test; TMT, Trials Making Test; VOT, Visual Organization Test; WAIS, Wechsler-Adult Intelligence Scale.

Comparing the two patient groups, aDM1 showed a more severe impairment of executive functions, as shown by the phonemic fluency (p = 0.001) and Trail Making Test A and B (p<0.001), reasoning and problem solving, as demonstrated by scores at the Raven matrices (p = 0.01), memory, as shown by scores at the Rey-Osterrieth complex figure recall (p = 0.01), visuospatial abilities, as demonstrated by scores at the Hooper VOT test (p = 0.03), and language, as shown by the Boston Naming Test (p = 0.04). aDM1 patients showed also higher HDRS (p = 0.02) and HARS (p = 0.04) scores compared with jDM1 cases. However, after adjusting for subject’s age, aDM1 relative to jDM1 patients showed more severe impairment only at the phonemic fluency (p = 0.006).

### MRI results

#### a) Conventional MRI findings

**Figure 1:**
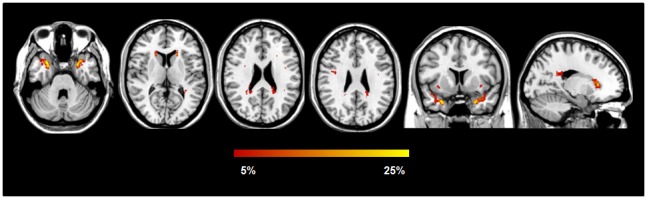
White matter hyperintensities spatial distribution in patients with myotonic dystrophy 1. The color scale (from 5% to 25%) represents the minimum to maximum probability of a lesion occurring in a particular spatial location. Results are overlaid on the coronal, sagittal and axial sections of the Montreal Neurological Institute standard brain in radiological convention (right is left).

White matter hyperintensities (WMHs) were found in 5/34 HC (15%) and 47/51 patients (92%), including 14 jDM1 (82%) and 33 aDM1 (97%). Wahlund scale score and WMH load were higher in DM1 patients relative to HC (Table 1). Wahlund scale score was also higher in aDM1 compared with jDM1 patients. At visual assessment, the areas most frequently involved were the frontal (47% of jDM1, 82% of aDM1), anterior temporal (59% of jDM1, 65% of aDM1), parieto-occipital (18% of jDM1, 35% of aDM1), and posterior (23% jDM1, 35% of aDM1) and anterior periventricular (29% of jDM1, 26% of aDM1) regions. Lesion probability maps analysis showed that the probability of lesions occurring in the same spatial location was 23.5% in the anterior temporal lobe bilaterally, 16% in the left and 12% in the right anterior periventricular WM, 10% in the left and 8% in the right inferior fronto-occipital fasciculus, 8% in the right superior longitudinal fasciculus (SLF), and 6% in the bilateral posterior periventricular WM and splenium of the corpus callosum (Figure 1). Other WM regions showed a voxel-wise WMH frequency lower than 5%.

#### b) VBM

**Figure 2:**
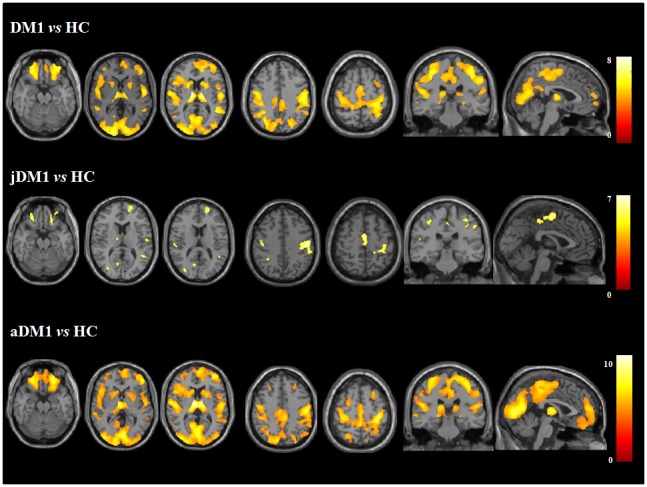
Voxel-based morphometry results in patients with myotonic dystrophy 1 compared with age-matched healthy controls adjusting for age and total intracranial volume. Regions of grey matter atrophy are shown in yellow-to-red and overlaid on the coronal, sagittal and axial sections of the Montreal Neurological Institute standard brain in radiological convention (right is left). Results are displayed at p<0.05 corrected for multiple comparisons.

Voxel-based morphometry (VBM) revealed a marked, almost symmetric, grey matter (GM) atrophy in DM1 patients compared to HC, including cortical and subcortical structures (Figure 2; Table S1). Most notably, pre- and post-central, supplementary motor (SMA), orbitofrontal, medial and dorsolateral frontal, insular, anterior and posterior cingulate, lateral and medial parietal, lateral temporal, hippocampal, and occipital cortical areas were affected bilaterally. Subcortical GM atrophy was also found in the thalamus, caudate nucleus, and putamen bilaterally. The pattern of GM atrophy in aDM1 patients compared with HC mirrored that of the whole DM1 group (Figure 2). On the contrary, GM atrophy in jDM1 patients relative to HC was less distributed compared with that of the whole group (Figure 2), showing only small regions of atrophy in the pre- and post-central gyri, SMA, orbitofrontal, dorsal frontal and lateral temporal cortices, parietal regions (>left), occipital cortices (>right) bilaterally, left cingulate cortex, and right thalamus. No significant differences were found comparing jDM1 and aDM1 patients. Group comparisons were performed also adjusting for years of education and the results did not change (Figure S1).

#### c) DT MRI

**Figure 3:**
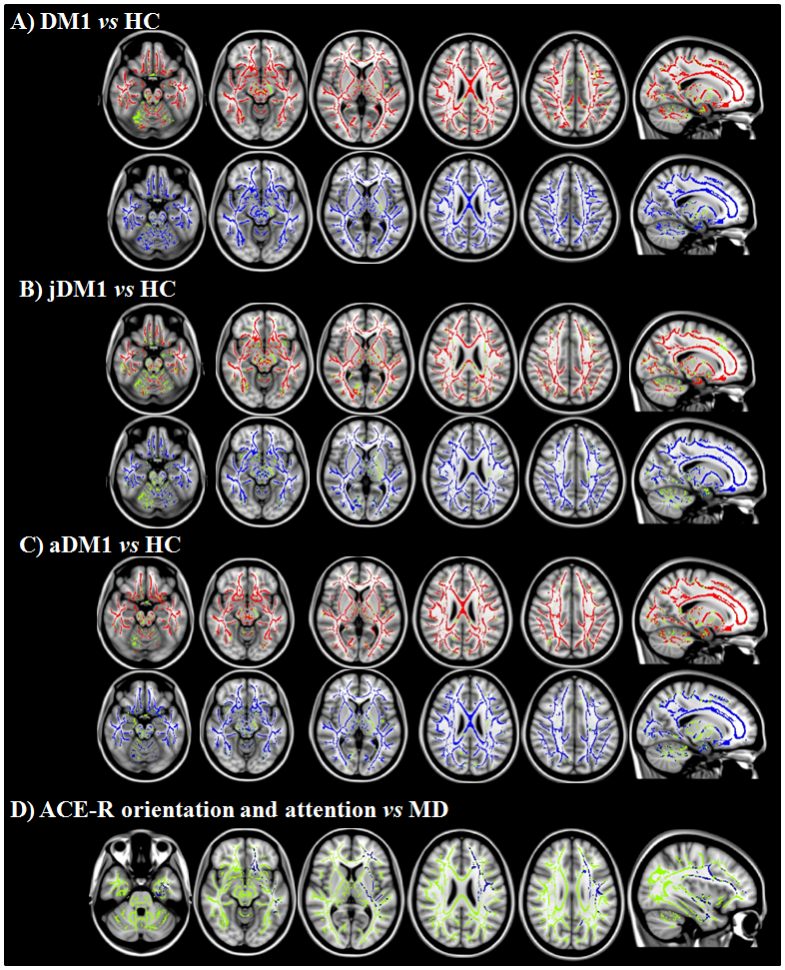
Tract-based spatial statistics results in patients with myotonic dystrophy 1 compared with age-matched healthy controls and relationship between the Addenbrooke’s Cognitive Examination–Revised (ACE-R) orientation and attention subscores and mean diffusivity values. Analyses were adjusted for age. A–C: Voxelwise group differences are shown in blue (mean diffusivity) and red (fractional anisotropy). D) Regions where MD values correlated with the ACE-R orientation and attention subscores are shown in blue. Results are overlaid on the sagittal and axial sections of the Montreal Neurological Institute standard brain in radiological convention (right is left), and displayed at p<0.05 corrected for multiple comparisons. The white matter skeleton is green.

Compared with HC, DM1 patients showed a distributed and symmetric pattern of fractional anisotropy (FA) decrease in the corpus callosum and the majority of association white matter (WM) tracts, including the SLF, inferior longitudinal (ILF), inferior fronto-occipital and uncinate fasciculi, fornices, and cingulum (Figure 3). In addition, projection fibres including internal (anterior/posterior limbs, retrolenticular parts) and external capsule were involved bilaterally (Figure 3). DM1 patients relative to HC showed FA decrease also in the brainstem. The areas of increased mean diffusivity (MD; Figure 3) and radial diffusivity (radD; Figure S2) closely mirrored those of decreased FA. No axial diffusivity (axD) differences were found between groups.

The results were similar when both aDM1 and jDM1 groups were compared with HC (Figure 3). The direct comparison between patient groups did not reveal any significant difference.

Group comparisons were performed also adjusting for years of education and the results did not change (Figure S3).

#### d) Correlations

In DM1 patients as a whole group, WMH load correlated with age (R = 0.40, p = 0.001) and disease duration (R = 0.42, p = 0.002). Moreover, WMH load was significantly associated with the scores obtained at the following neuropsychological tests: Addenbrooke’s Cognitive Examination–Revised (ACE-R) total (R = −0.31, p = 0.047), ACE-R memory subtest (R = −0.42, p = 0.005), Rey Auditory Verbal Learning test (RAVLT) total (R = −0.33, p = 0.02), RAVLT recognition (R = −0.31, p = 0.03), Raven matrices (R = −0.39, p = 0.01), Wisconsin card sorting test (WCST) categories (R = −0.39, p = 0.01), and Hooper VOT (R = −0.32, p = 0.04). WMH load was also associated with HDRS score (R = 0.28, p = 0.04). Adjusting for subject’s age, WMH load correlated only with scores at the ACE-R memory subtest (R = −0.32, p = 0.04) and Raven matrices (R = −0.34, p = 0.03). DSS score was not associated with cognitive tests.

No correlation was found between VBM findings and other variables. TBSS analysis showed a negative correlation between the ACE-R orientation and attention subscores and MD values of the left corona radiata, internal and external capsule, and frontotemporal WM regions belonging to the inferior fronto-occipital/uncinate fasciculi, ILF and SLF (Figure 3). No other correlations were found between WM microstructural damage and clinical and cognitive features, including CGT repeats and DSS score. DT MRI measures did not correlate with WMH load.

## Discussion

We present clinical, cognitive and structural MRI data in a large series of patients with noncongenital DM1. Our DM1 patients showed a moderate muscular impairment. Sleepiness and fatigue occurred in about half of them. An impaired cognitive performance over a broad range of functions, including frontal, visuospatial, naming and memory abilities, was also observed. MRI showed that DM1 patients had altered structural brain measures relative to controls including regional GM volumes, WMHs, and WM microstructural tract damage. WM abnormalities, but not GM involvement, correlated with the severity of cognitive deficits.

VBM showed a severe and distributed pattern of GM atrophy including the key nodes of the sensorimotor and main cognitive brain networks. The involvement of cortical and subcortical regions in DM1 is in agreement with pathological studies documenting cell loss, intracellular NFT or eosinophilic intranuclear inclusions in neurons of the cerebral cortex, mostly in the frontal and temporal lobes, thalami, basal ganglia and brainstem [3]–[5]. Our data also agree with previous, preliminary MRI studies in smaller DM1 populations [15]–[19]. Primary sensorimotor cortex, SMA, thalamic and basal ganglia atrophy can contribute to the impaired motor planning and execution known to occur in these patients. A correlation between motor areas volumes and CTG triplet expansion has been previously reported [17]. In addition, a functional MRI study showed an altered pattern of motor system activations in DM1 patients during a motor task [38]. In our study, DM1 patients also showed a severe involvement of the dorsolateral prefrontal cortex, cingulum, medial and lateral parietal regions, occipital and temporal lobes, that has been associated with cognitive deficits and personality changes in many neurological and psychiatric diseases. Similar symptoms, such as mild depression and anxiety as well as deficits of visuospatial abilities, executive functions, reasoning and naming, have been frequently reported in DM1 patients [6], [25], [39], [40], including those of our study.

We demonstrated a high prevalence of WMHs in DM1 patients, which increased with age and disease duration. Visual assessment and lesion probability maps showed that WMHs were mainly located in the anterior temporal, frontal, parieto-occipital and periventricular WM regions. Several MRI studies have shown that DM1 patients have WMHs, which are generally more prominent in the frontal, parietal and temporal regions and increase with the progression of the disease [7], [10]–[13], [15], [41]–[43]. However, they used qualitative scales to evaluate the spatial distribution of WMHs. In this study, by creating lesion probability maps, we provide a quantitative assessment of the anatomic location of WMHs and show that they are mainly located in areas relevant for cognition. In addition, our DM1 patients experienced a severe and distributed WM microstructural damage, which extends well beyond focal WMHs and includes interhemispheric, corticospinal, limbic and associative pathways. Postmortem studies of DM1 indicated that a disordered arrangement of myelin sheaths/axons, fibrillar gliosis, increased interfascicular and perivascular spaces are likely to be the main substrates of WMHs and DT MRI findings in these patients [5], [6], [41]. Myelin alterations and changes in interaxonal water content may also explain the increased T2 relaxation times [44] and decreased magnetization transfer ratio of the normal appearing WM [45] observed in DM1 patients.

In our DM1 patients, the severity of cognitive deficits correlated with WM damage, in terms of both WMHs and microstructural damage, but not with GM atrophy. In particular, we found that WMH burden was associated with memory, executive, reasoning, and visuospatial impairments. Furthermore, an association was observed between orientation and attention deficits and the microstructural abnormalities detected in the internal capsule and major associative WM tracts linking the frontal and temporal lobes. Previous studies suggested a relationship between the extent of WMHs and cognitive deficits in DM1 patients [6], [7], [10], [12], [13], [15], although other studies did not confirm these findings [8], [17], [42]. In addition, in aDM1 patients DT MRI findings have been related to disease duration, CTG repeat expansion sizes, MIRS score, manual motor performance [18] and facial muscle volumes [19], but not with cognitive impairment [18]. A correlation between WM damage and intelligence tests was observed in congenital and jDM1 [20]. The conflicting results between our and previous studies concerning the correlation of structural brain abnormalities, clinical features and cognitive/behavioural findings can be attributed mainly to the high variability of the DM1 clinical presentation based on the somatic mosaicism of CTG repeats [2], but also to the small and heterogeneous cohorts of patients involved in previous reports.

A significant number of our DM1 cases had mild to moderate sleepiness. It has been suggested that that excessive daytime sleepiness in DM1 is primarily caused by central mechanisms, i.e., damage to brainstem regions responsible for sleep control [46]–[49]. TBSS analysis showed a severe damage to brainstem regions in both jDM1 and aDM1 groups, although we did not find a correlation between the DSS score and WM findings. Peripheral mechanisms can play a significant role but mainly in the most severely affected patients with oropharyngeal and respiratory weakness who develop sleep apnoea and night hypoventilations [50]. However, in our sample, only a few patients experienced a moderate respiratory failure syndrome, with no relationship between DSS score and FVC.

Finally, in this study we assessed brain GM and WM damage separately in aDM1 and jDM1 patients. It is worth noting that the classification of DM1 patients based on the age of symptom onset is a challenging and somewhat arbitrary task. To date, however, there are no established recommendations for this stratification. Some authors suggested that symptoms of juvenile DM1 begin after 1 month of life and before the age of 10 years [1], [51], others indicated that juvenile DM1 is defined by onset before 16 years of age [52]. In the present and in two previous studies [25], [53], we defined juvenile DM1 cases those subjects with symptom onset before the age of 20 years, since such a cut-off is often used for dividing juvenile and adult forms in different neurological disorders, for instance in parkinsonism. In 2000, the International Myotonic Dystrophy Consortium [54] suggested that the age at onset and disease severity of DM1 significantly correlate with the number of CGT repeats. However, the same panel also indicated that the “use of these ranges in predicting age at onset or clinical severity in individual patients can be misleading because of the large overlap between phenotypic classes and the somatic mosaicism of CTG repeat alleles”. A clear demonstration of such a poor genotype-phenotype relationship is the lack of correlation between brain GM and WM damage and number of CTG repeats in our DM1 patients, regardless of the age at onset. Although we cannot exclude that a different definition of patient subgroups may have led to different findings, the assessment of brain damage in aDM1 and jDM1 patients with similar disease duration, CTG repeats and muscular involvement allowed us to speculate on the possible separated effects of disease itself and aging. aDM1 patients showed equivalent, severe and distributed patterns of cortical and subcortical GM atrophy and WM tract damage, while in jDM1 patients WM microstructural damage extensively exceeded GM involvement. Although the cross-sectional nature of our study does not allow to conclude on the neurodegenerative and/or developmental origin of the brain abnormalities observed in DM1 [55], our findings suggest a strong effect of age on GM, thus supporting the hypothesis of a degenerative process (premature aging) for the GM abnormalities found in this condition. In contrast, the severe and distributed WM microstructural damage observed in both DM1 patient groups (i.e., regardless of age) might be interpreted as a result of developmental changes. Longitudinal studies in patients involved at disease onset are warranted to confirm these hypotheses.

The study is not without limitations. First, healthy controls did not perform neuropsychological evaluation. Second, the two patient groups were different in size. This could have, at least partially, influence the statistical analysis. Third, we did not investigate social cognition and other psychiatric features, which have been demonstrated to be more altered in jDM1 relative to aDM1 patients [56]. Finally, due to the exploratory nature of the study, correlation analysis between WMH load and cognitive variables was not corrected for multiple comparisons.

Overall these results suggest that DM1 is characterized by a severe brain involvement. The relationship between WM damage and cognitive deficits supports the notion of a structural (and functional) multiple disconnection between GM structures, secondary to WM tract breakdown, as one of the most important factors leading to cognitive impairment in DM1. With some caution related to the DM1 stratification based on age of symptom onset, MRI findings in aDM1 and jDM1 patients may shed light into the different pathogenetic mechanisms underlying brain damage in this multisystem disease and contribute to identify new therapeutic targets.

## Supporting Information

Figure S1 **Voxel-based morphometry results in patients with myotonic dystrophy 1 compared with age-matched healthy controls, adjusting for age, total intracranial volume and years of education.**
(DOCX)

Figure S2 **Radial diffusivity results in patients with myotonic dystrophy 1 compared with age-matched healthy controls.**
(DOCX)

Figure S3 **Tract-based spatial statistics results in patients with myotonic dystrophy 1 compared with age-matched healthy controls.** Analyses were adjusted for age and years of education.
(DOCX)

Table S1 **Voxel-based morphometry results.**
(DOC)

Appendix S1 **Detailed description of MRI acquisition and analysis methods.**
(DOCX)
