# Supplementary material for: Cognitive Impairment in Myotonic Dystrophy Type 1 Is Associated with White Matter Damage
Source: PLoS One. 2014 Aug 12;9(8):e104697. doi: 10.1371/journal.pone.0104697 (PMC4130603; doi:10.1371/journal.pone.0104697)
Supplement: Figure S1 — Voxel-based morphometry results in patients with myotonic dystrophy 1 compared with age-matched healthy controls, adjusting for age, total intracranial volume and years of education. (DOCX) [file pone.0104697.s001.docx]

**Figure S1.** Voxel-based morphometry results in patients with myotonic dystrophy 1 compared with age-matched healthy controls, adjusting for age, total intracranial volume and years of education. Regions of grey matter atrophy are shown in yellow-to-red and overlaid on the coronal, sagittal and axial sections of the Montreal Neurological Institute standard brain in radiological convention (right is left). Results are displayed at p<0.05 corrected for multiple comparisons.

**
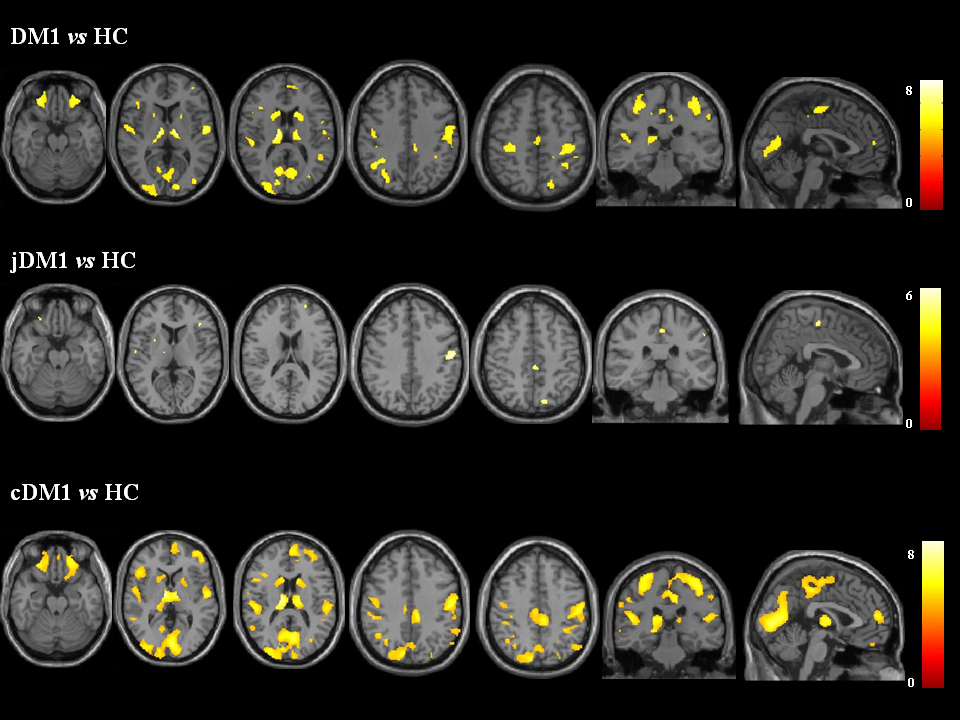
**
