# Supplementary material for: Cognitive Impairment in Myotonic Dystrophy Type 1 Is Associated with White Matter Damage
Source: PLoS One. 2014 Aug 12;9(8):e104697. doi: 10.1371/journal.pone.0104697 (PMC4130603; doi:10.1371/journal.pone.0104697)
Supplement: Figure S2 — Radial diffusivity results in patients with myotonic dystrophy 1 compared with age-matched healthy controls. (DOCX) [file pone.0104697.s002.docx]

**Figure S2.** Radial diffusivity results in patients with myotonic dystrophy 1 compared with age-matched healthy controls. Voxelwise group differences are shown in yellow. Results are overlaid on the sagittal and axial sections of the Montreal Neurological Institute standard brain in radiological convention (right is left), and displayed at p<0.05 corrected for multiple comparisons. The white matter skeleton is green.

**
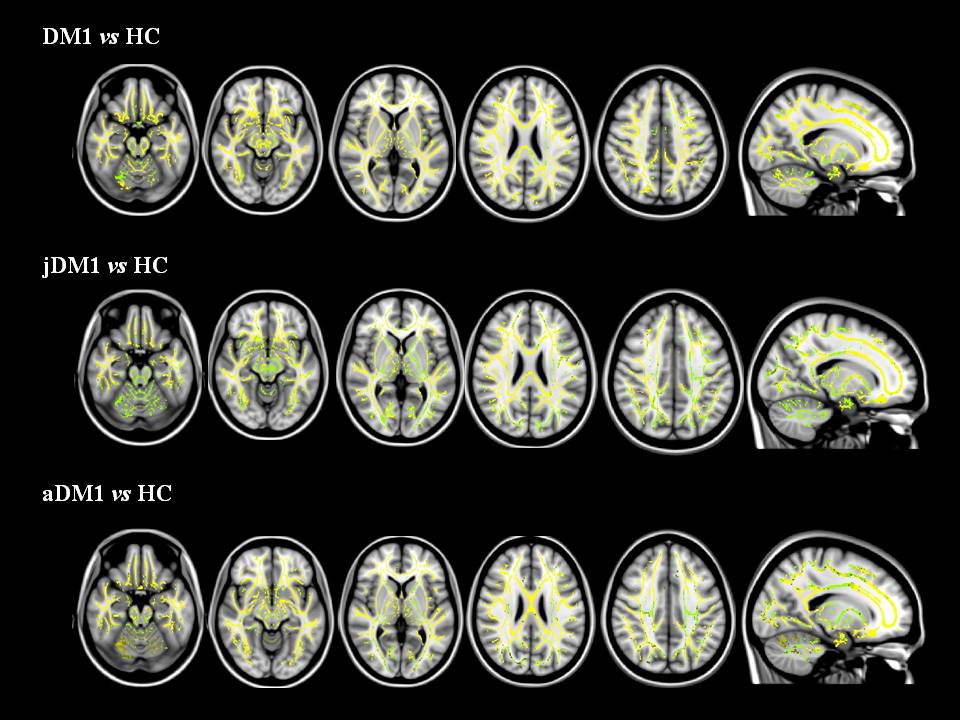
**
