# Supplementary material for: Cognitive Impairment in Myotonic Dystrophy Type 1 Is Associated with White Matter Damage
Source: PLoS One. 2014 Aug 12;9(8):e104697. doi: 10.1371/journal.pone.0104697 (PMC4130603; doi:10.1371/journal.pone.0104697)

**Figure S3.** Tract-based spatial statistics results in patients with myotonic dystrophy 1 compared with age-matched healthy controls. Analyses were adjusted for age and years of education. Voxelwise group differences are shown in blue (mean diffusivity) and red (fractional anisotropy). Results are overlaid on the sagittal and axial sections of the Montreal Neurological Institute standard brain in radiological convention (right is left), and displayed at p<0.05 corrected for multiple comparisons. The white matter skeleton is green.


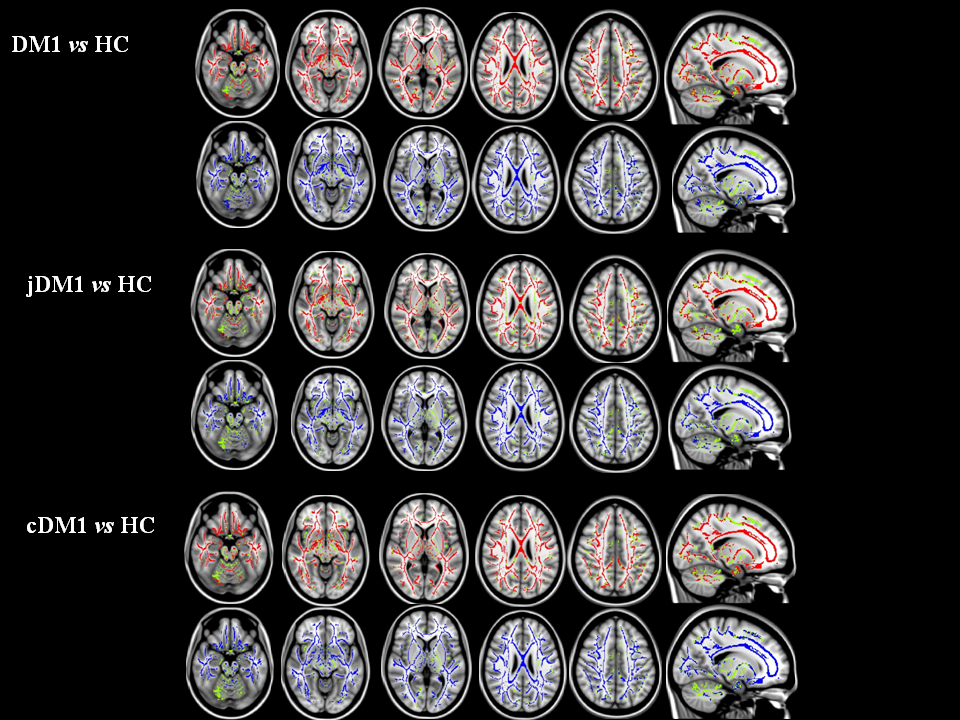

Supplement: Figure S3 — Tract-based spatial statistics results in patients with myotonic dystrophy 1 compared with age-matched healthy controls. Analyses were adjusted for age and years of education. (DOCX) [file pone.0104697.s003.docx]
