# Supplementary material for: Cognitive Impairment in Myotonic Dystrophy Type 1 Is Associated with White Matter Damage
Source: PLoS One. 2014 Aug 12;9(8):e104697. doi: 10.1371/journal.pone.0104697 (PMC4130603; doi:10.1371/journal.pone.0104697)
Supplement: Table S1 — Voxel-based morphometry results. (DOC) [file pone.0104697.s004.doc]

**Table S1.** Voxel-based morphometry results of grey matter atrophy in DM1 whole group, jDM1 and aDM1 patients compared to HC (p < 0.05, Family-wise error corrected).

| **DM1 whole group *vs* HC** | | | | |
| --- | --- | --- | --- | --- |
| Brain area | Brodmann | Coordinates | T value | Cluster (ke) |
| L precentral gyrus | 4 | -41, -18, 45 | 5.61 | 45.599 |
|  | 6 | -40, -13, 50 | 4.84 |  |
| R precentral gyrus | 4 | 47, -7, 39 | 6.35 |  |
|  | 6 | 39, -15, 50 | 5.49 |  |
|  | 6 | 49, -3, 51 | 5.03 |  |
| R central sulcus | 4 | 58, -5, 31 | 6.82 |  |
| L SMA | 6 | -3,-12, 50 | 5.04 |  |
| R SMA | 6 | 3, -12, 54 | 5.92 |  |
| L postcentral gyrus | 3 | -31, -34, 58 | 8.07 |  |
|  |  | -52, -16, 37 | 7.75 |  |
|  |  | -45, -22, 42 | 7.45 |  |
| R postcentral gyrus | 3 | 36, -29, 53 | 7.32 |  |
|  |  | 47, -17, 37 | 7.09 |  |
| L angular gyrus | 39 | -37, -62, 48 | 4.85 |  |
| L supramarginal gyrus | 40 | -48, -30, 36 | 5.83 |  |
| R supramarginal gyrus | 40 | 48, -30, 46 | 4.99 |  |
| L SPL | 7 | -18, -74, 47 | 5.66 |  |
| R SPL | 7 | 22, -65, 49 | 5.10 |  |
| L IPL | 40 | -56, -32, 42 | 6.04 |  |
| R IPL | 39 | 43, -53, 39 | 7.00 |  |
| L STG | 48 | -55, -8, 5 | 7.64 |  |
|  |  | -44, -43, 12 | 7.13 |  |
| R STG | 48 | 59, -9, 10 | 7.35 |  |
| L MTG | 21 | -46, -51, 14 | 7.55 |  |
| L SOG | 7 | -21, -74, 43 | 6.92 |  |
|  |  | -13, -89, -3 | 6.05 |  |
| R SOG | 18 | 21, -88, 25 | 8.40 |  |
|  |  | 23, -74, 23 | 7.45 |  |
| L MOG | 19 | -25, -81, 23 | 9.28 |  |
|  |  | -13, -94, 2 | 7.40 |  |
| R MOG | 18 | 29, -73, 28 | 7.34 |  |
|  |  | 28, -96, 13 | 7.32 |  |
|  |  | 29, -89, 16 | 7.27 |  |
|  |  | 34, -92, 6 | 7.12 |  |
|  |  | 28, -79, 27 | 7.05 |  |
| L calcarine cortex | 18 | -3, -68, 16 | 7.14 |  |
| R calcarine cortex | 18 | 18, -94, 2 | 7.70 |  |
|  |  | 21, -75, -7 | 7.69 |  |
|  |  | 17, -82, 14 | 7.44 |  |
|  |  | 14, -69, 14 | 7.25 |  |
| R cuneus | 19 | 18, -79, 23 | 7.08 |  |
| R caudate | - | 14, 12, 19 | 7.00 |  |
| L thalamus | - | -11,-11,10 | 9.26 | 1921 |
| R thalamus | - | 13, -11, 10 | 9.87 |  |
| R hippocampus | - | 23, -26, -4 | 5.69 |  |
| L inferior orbitofrontal cortex | 11 | -20, 24, -17 | 6.56 | 11389 |
|  |  | -32, 34, -6 | 6.09 |  |
| R inferior orbitofrontal cortex | 11 | 30, 38, -22 | 7.96 |  |
|  | 47 | 36, 42, -5 | 5.67 |  |
| L middle orbitofrontal cortex | 11 | -20, 43, -24 | 7.73 |  |
|  |  | -23, 38, -24 | 7.65 |  |
|  |  | -28, 44, -21 | 7.58 |  |
|  |  | -29, 47, -19 | 7.45 |  |
|  |  | -29, 42, -8 | 7.38 |  |
| L superior frontal orbital cortex | 11 | -14, 59, -5 | 5.51 |  |
| R superior orbitofrontal cortex | 11 | 19, 28, -15 | 6.69 |  |
|  |  | 18, 51, -10 | 5.47 |  |
|  |  | 18, 46, -12 | 5.46 |  |
| L SFG | 10 | -12, 54, 1 | 5.40 |  |
| R SFG | 32 | 13, 50, 31 | 5.50 |  |
|  |  | -15, 52, 11 | 6.89 |  |
|  |  | -20, 55, 11 | 6.06 |  |
| L MFG | 10 | -35, 50, 8 | 6.77 |  |
|  |  | -31, 58, 13 | 6.22 |  |
|  | 46 | -32, 45, 15 | 5.76 |  |
|  |  | -29, 43, 20 | 5.68 |  |
|  |  | -31, 43, 33 | 5.31 |  |
| R MFG | 46 | 22, 50, 27 | 6.03 |  |
|  |  | 27, 38, 24 | 6.63 |  |
| L IFG-pt | 45 | -45, 42, 1 | 6.44 |  |
|  |  | -39, 42, 13 | 5.51 |  |
| L ACC | 32 | -11, 43, 13 | 6.36 |  |
|  |  | -1, 53, 13 | 6.12 |  |
| L caudate | - | -11, 13, 15 | 6.74 |  |
| L putamen | - | -20, 9, 12 | 6.39 |  |
| R MFG | 6 | 25, -2, 53 | 5.57 | 118 |
| L IFG-po | 44 | -36, 14, 33 | 5.32 | 117 |
|  |  | -36, 5, 28 | 5.07 |  |
| L rectus gyrus | 11 | -3, 49, -17 | 5.27 | 504 |
| R SPG | 7 | 26, -66, 60 | 5.19 | 29 |
| R MTG | 37 | 53, -62, 11 | 5.20 | 101 |
|  |  | 48, -49, 12 | 4.88 |  |
| L ITG | 37 | -50, -61, -9 | 6.13 | 154 |
| L fusiform gyrus | 37 | -36, -49, -9 | 5.59 | 171 |
| L lingual gyrus | 19 | -25, -57, -7 | 5.25 |  |
| R insula | 48 | 45, 3, -31 | 5.43 | 25 |
| L hippocampus | - | -24, -26, -4 | 5.78 | 43 |
| **jDM1 *vs* HC** | | | | |
| L precentral gyrus | 6 | -40, -13, 45 | 6.01 | 1287 |
|  | 6 | -35, -14, 42 | 5.63 |  |
|  | 4 | -34, -22, 56 | 5.75 |  |
| R precentral gyrus | 6 | 49, -9, 40 | 5.40 | 207 |
|  | 4 | 52, -8, 40 | 5.32 |  |
| L SMA | 6 | -1, -12, 54 | 5.52 | 340 |
| R SMA | 6 | 3, -12, 54 | 5.74 |  |
| L postcentral gyrus | 3 | -50, -17, 40 | 7.57 | 1287 |
|  |  | -41, -24, 42 | 6.60 |  |
|  |  | -33, -33, 55 | 6.40 |  |
|  |  | -33, -23, 53 | 6.34 |  |
|  |  | -56, -32, 42 | 6.30 |  |
| R postcentral gyrus | 3 | 49, -15, 35 | 5.93 | 207 |
| L SFG | 48 | 28, 36, -20 | 6.59 | 282 |
| R SFG | 6 | 19, 40, 22 | 6.25 | 179 |
| L MFG | 18 | 36, -79, 14 | 6.27 | 66 |
| R MFG | 46 | 27, 38, 22 | 7.63 | 179 |
| L rectus gyrus | 24 | 34, -29, 51 | 5.94 | 325 |
| L superior frontal orbital gyrus |  | 33, -48, 37 | 5.87 |  |
| L ACC | 3 | 59, -21, 16 | 5.83 | 71 |
| L MCC | 48 | 13, -11, 8 | 5.74 |  |
| L IFG-po | 11 | 8, -11, 10 | 11.43 | 26 |
| R middle frontal orbital cortex | 10 | -46,-51, 12 | 6.40 | 142 |
| L middle inferior orbital cortex |  | -9,-10, 10 | 10.02 | 23 |
| L IPL | 3 | 55, -11, 28 | 5.98 | 1287 |
| R IPL | 42 | 59, -9, 10 | 5.79 | 46 |
| L SPL | 6 | -2, -12, 56 | 5.73 | 21 |
| L precuneus | 3 | -18, -74, 46 | 5.76 | 1287 |
| L STG | 11 | -34, 50, 6 | 6.49 | 133 |
|  |  | -15, -94, 2 | 6.42 |  |
| R STG | 48 | 29, -75, 32 | 5.67 | 26 |
|  |  | -43, 39, -1 | 5.64 |  |
| L MTG | 19 | -12, 42, -15 | 5.96 | 54 |
| L MOG | 37 | 12, -69, 14 | 6.24 | 75 |
| R MOG | 47 | -43, 39, -1 | 5.64 | 45 |
| R calcarine cortex | 11 | -3, 39, -12 | 5.76 | 48 |
| R lingual gyrus | 18 | 21, -73, -12 | 7.65 | 303 |
| R thalamus | 19 | -31, 44, -10 | 5.58 | 45 |
| **aDM1 *vs* HC** | | | | |
| L precentral gyrus | 4 | -41, -18, 45 | 6.75 | 75346 |
|  | 6 | -40, -13, 50 | 5.20 |  |
| R precentral gyrus | 4 | 47, -7, 39 | 6.94 |  |
|  | 6 | 39, -15, 50 | 6.38 |  |
|  | 10 | -22, 38, -20 | 8.29 |  |
| L SMA | 6 | -1, -13, 54 | 5.63 |  |
| R SMA | 6 | 3, -12, 54 | 5.57 |  |
| L postcentral gyrus | 3 | -2, -8, 56 | 6.21 |  |
| R postcentral gyrus | 6 | -37, -62, -48 | 5.57 |  |
| L MFG | 11 | 30, 38, -20 | 8.42 |  |
| L superior frontal orbital cortex | 45 | -46, 43, 1 | 8.09 |  |
| L middle frontal orbital gyrus |  | 50, 16, 10 | 7.73 |  |
| R inferior frontal orbital gyrus |  | -3, 60, 9 | 7.84 |  |
| L IFG-pt | 48 | -1, 55, 13 | 7.76 |  |
| L IFG-po | 10 | 23, -89, 25 | 8.97 |  |
| L frontomedial cortex | 18 | -25,-81, 23 | 8.28 |  |
| L angular gyrus | 39 | 48, -30, 46 | 5.52 |  |
| R angular gyrus | 40 | -18, -74, 46 | 5.83 |  |
| L supramarginal gyrus | 40 | 22, -65, 51 | 5.40 |  |
| R supramarginal gyrus | 7 | -56, -32, 42 | 5.97 |  |
| L SPL | 7 | 43, -30, 46 | 5.67 |  |
| R SPL | 40 | -48, -19, 42 | 9.21 |  |
| L IPL | 40 | 37, -24, 65 | 7.73 |  |
| R IPL | 4 | -35, 50, 10 | 8.96 |  |
| L STG | 19 | 24, -1, 56 | 6.55 |  |
| R STG | 48 | 14, -80, 41 | 7.83 |  |
| R SOG | 19 | 40, -89, 5 | 7.88 |  |
| L MOG | 18 | 14, -62, 9 | 7.97 |  |
| R MOG | 19 | 35, -90, 6 | 7.95 |  |
|  | 18 | -21, -75, 20 | 7.83 |  |
|  | 18 | 23, -75, -11 | 8.26 |  |
|  | 19 | 18, -94, 2 | 8.06 |  |
| R fusiform gyrus | 18 | 14, -84, 14 | 7.78 |  |
| L calcarine cortex | 17 | -6, -31, 67 | 8.43 |  |
|  | 48 | 5, -16, 10 | 8.09 |  |
| R calcarine cortex | 17 | 12, -86, 18 | 7.76 |  |
|  | 19 | -3, -68, 16 | 8.75 |  |
|  | 17 | -10, -95, 2 | 7.80 |  |
|  | 18 | -36, -24, 56 | 8.58 |  |
| R cuneus | 6 | 54, -59, 14 | 6.39 |  |
| R thalamus | 22 | -53, -23, 12 | 7.94 |  |
| L thalamus |  | 42,-24, 53 | 5.89 | 3021 |
| L SFG | 8 | -28, -54, -13 | 6.12 | 276 |
|  | 37 | -39, 26, 29 | 6.03 |  |
| R MFG | 44 | -50, -61, -9 | 5.59 | 48 |
| L IFG-pt | 6 | -22, -27, -4 | 5.63 | 31 |
| R MTG | 37 | 28, 9, 47 | 5.70 | 340 |
| L ITG | 37 | -50, -61, -9 | 5.59 | 31 |
| L lingual gyrus | 37 | -24, -51, -48 | 5.54 | 139 |
| L fusiform gyrus | 48 | -40, 14, 35 | 5.69 | 290 |
| L hippocampus | - | -22, -27, -4 | 5.63 | 31 |
| R hippocampus | 3 | -2, -8, 56 | 5.78 | 3021 |

**Abbreviations:** ACC, anterior cingulate cortex;. aDM1, Myotonic dystrophy 1 with adult onset (≥ 20 years); DM1, Myotonic dystrophy type 1; HC, healthy controls; IFG, inferior frontal gyrus; IFG-po, inferior frontal gyrus pars opercularis; IFG-pt, inferior frontal gyrus pars triangularis; ITG, inferior temporal gyrus; IPL, inferior parietal lobule; jDM1, Myotonic dystrophy type 1 with childhood onset (< 20 years); L, left; R, right; MCC, middle cingulate cortex; MFG, middle frontal gyrus; MOG, middle occipital gyrus; MTG, middle temporal gyrus; SFG, superior frontal gyrus; SOG, superior occipital gyrus; SPL, superior parietal lobule; SMA, supplementary motor area; STG, superior temporal gyrus.
