# Supplementary material for: Cognitive Impairment in Myotonic Dystrophy Type 1 Is Associated with White Matter Damage
Source: PLoS One. 2014 Aug 12;9(8):e104697. doi: 10.1371/journal.pone.0104697 (PMC4130603; doi:10.1371/journal.pone.0104697)
Supplement: Appendix S1 — Detailed description of MRI acquisition and analysis methods. (DOCX) [file pone.0104697.s005.docx]

**MRI acquisition**

MRI scans were acquired using a 1.5 T system (Philips Medical Systems, Achieva). The following sequences were acquired: (i) dual-echo (DE) turbo spin-echo (SE) (repetition time [TR]=3125 ms, echo time [TE]=20/100 ms, echo train length=6, 44 axial slices, thickness=3.0 mm, matrix size =256×256, field of view [FOV]=240 mm^2^); (ii) three-dimensional (3D) T1 transient field echo (phase direction=anterior-posterior; TR=7.34 ms, TE=3.398 ms, inversion time= 1000 ms, flip angle=8^°^, matrix size=256×256×180, FOV=256×256×180 mm); and (iii) pulsed gradient SE single shot echo-planar (TR=6713, TE=86, flip angle=90°, matrix size=112 × 112, FOV=224 mm^2^; 50 contiguous, 2.6-mm thick, axial slices), with diffusion-encoding gradients applied in 65 non-collinear directions (b factor= 1000 s/mm^2^ and seven b0 volumes).

**MRI analysis**

All MRI post-processing was performed by a single experienced observer, blinded to subject’s identity.

*a) Conventional MRI*

WMHs, if any, were identified on the DE scans. WMH severity was graded according to the age-related WM change scale (Wahlund scale) [1]. In our lab, inter- and intra-observer reproducibilities of this scale are greater than 0.90 (data not shown). WMH load was measured by a semi-automatic threshold-based approach using the Jim software package (Version 5.0, Xinapse Systems, Northants, UK, <http://www.xinapse.com>). WMH masks were created in the DE space and transformed into the Montreal Neurological Institute (MNI) space using the rigid transformations calculated between the T2-weighted and T1-weighted images and those calculated for the voxel-based morphometry (VBM) analysis (see next paragraph). After averaging, a map containing information about the degree of occurrence of lesions at a voxel level was produced. This map was used to assess the spatial distribution of WMHs.

*b) VBM*

VBM was performed using SPM8 (Statistical Parametric Mapping, Wellcome Department of Imaging Neuroscience, London, UK) and the Diffeomorphic Anatomical Registration using Exponentiated Lie algebra (DARTEL) registration method [2]. Briefly, (i) T1-weighted images were segmented using the standard unified segmentation model[3] to produce grey matter (GM), white matter (WM) and cerebrospinal fluid (CSF) probability maps, (ii) the images were imported in DARTEL, rigidly aligned, and segmented into GM, WM and cerebrospinal fluid (CSF) (using the segmentation parameters from step [i]) and resampled to 1.5 mm isotropic voxels; (iii) GM segments were coregistered simultaneously using the fast diffeomorphic image registration algorithm [2]; (iv) the flow fields were then applied to the rigidly-aligned segments to warp them to the common DARTEL space and then modulated using the Jacobian determinants; (v) the modulated images from DARTEL were normalized to the MNI template using an affine transformation estimated from the DARTEL GM template and the *a priori* GM probability map without resampling ([http://brainmap.wisc.edu/normalizeDARTELtoMontreal Neurological Institute](http://brainmap.wisc.edu/normalizeDARTELtoMNI)). Prior to the statistical computations, the images were smoothed with an 8 mm Full-Width Half-Maximum Gaussian filter. The unsmoothed, modulated GM, WM and CSF images were used to calculate total intracranial volume.

*c) DT MRI*

DWI images were corrected for movements and distortions caused by eddy currents, using an implementation of an algorithm previously described [4] (<http://white.stanford.edu/newlm/index.php/DTI_Preprocessing#dti Raw_Preprocessing_Pipeline>). This eddy-current/motion correction step combines a rigid-body transformation (6 parameters) with a constrained non-linear warping (8 parameters) based on a model of the expected eddy-current distortions Additionally, an affine transformation was calculated and combined with the previous ones to transform DWI to the MNI space. The rotation component of the transformation was applied to the gradient vectors. The DT was estimated on a voxel-by-voxel basis using the DTIfit toolbox, part of the FMRIB Diffusion Toolbox within FSLv4.1.7 (<http://www.fmrib.ox.ac.uk/fsl/>) in order to obtain mean diffusivity (MD) and fractional anisotropy (FA) maps. Maps of axial diffusivity (axD) and radial diffusivity (radD) were also calculated [5]. Tract-based spatial statistics version 1.2 (<http://www.fmrib.ox.ac.uk/fsl/tbss/> index.html) was used to perform the multisubject DT MRI analysis [5]. FA volumes were aligned to the standard space using the following procedure: (i) the FMRIB58 FA standard space image was selected as the target FA image, (ii) the nonlinear transformation that mapped each subject’s FA to the target image was computed using FNIRT, (iii) the target image was transformed affinely to the MNI 152 standard space, and (iv) the same transformation was used to align each subject’s FA map to the standard space. A mean FA image was then created by averaging the aligned individual FA images and thinned to create a FA skeleton representing WM tracts common to all subjects [5]. The FA skeleton was thresholded at a value of 0.2 to exclude voxels with low FA values, which are likely to include GM or CSF. Individual MD, FA, axD, and radD data were projected onto this common skeleton. Two WM atlases within FSL (<http://fsl.fmrib.ox.ac.uk/fsl/data/atlas-descriptions.html>), the Johns Hopkins University WM tractography atlas and the ICBM-DTI WM labels atlas, guided the identification of WM tracts.

**References**

1. Wahlund LO, Barkhof F, Fazekas F, Bronge L, Augustin M, et al. (2001) A new rating scale for age-related white matter changes applicable to MRI and CT. Stroke 32: 1318-1322.

2. Ashburner J (2007) A fast diffeomorphic image registration algorithm. NeuroImage 38: 95-113.

3. Ashburner J, Friston KJ (2005) Unified segmentation. NeuroImage 26: 839-851.

4. Rohde GK, Barnett AS, Basser PJ, Marenco S, Pierpaoli C (2004) Comprehensive approach for correction of motion and distortion in diffusion-weighted MRI. Magnetic resonance in medicine : official journal of the Society of Magnetic Resonance in Medicine / Society of Magnetic Resonance in Medicine 51: 103-114.

5. Smith SM, Jenkinson M, Johansen-Berg H, Rueckert D, Nichols TE, et al. (2006) Tract-based spatial statistics: voxelwise analysis of multi-subject diffusion data. NeuroImage 31: 1487-1505.
